# Reverse end-diastolic flow in a fetus with a rare liver malformation: a case report

**DOI:** 10.1186/1752-1947-5-37

**Published:** 2011-01-27

**Authors:** Antonella Giancotti, Antonella Spagnuolo, Valentina D'Ambrosio, Gaia Pasquali, Brunella Muto, Francescantonio Bisogni, Renato La Torre, Maurizio Marco Anceschi, Fabrizio De Gado

**Affiliations:** 1 Department of Obstetrics and Gynecology, "Sapienza" University Umberto I Hospital, Viale del Policlinico, 155, 00161 Rome, Italy

## Abstract

**Introduction:**

We describe a case of early and persistent reverse end-diastolic flow in the middle cerebral artery in a fetus with severe ascites. These features are associated with a rare liver malformation known as ductal plate malformation.

**Case presentation:**

A 28-year-old Caucasian woman was referred to our high-risk obstetric unit at 24 weeks' gestation for fetal ascites detected during a routine ultrasound examination. During her hospitalization we performed medical investigations, including a fetal paracentesis, to detect the etiology of fetal ascites. The cause of fetal ascites (then considered non-immune or idiopathic) was not evident, but a subsequent ultrasound examination at 27 weeks' gestation showed a reverse end-diastolic flow in the middle cerebral artery without any other Doppler abnormalities. A cesarean section was performed at 28 weeks' gestation because of the compromised fetal condition. An autopsy revealed a rare malformation of intrahepatic bile ducts known as ductal plate malformation.

**Conclusion:**

Persistent reverse flow in the middle cerebral artery should be considered a marker of adverse pregnancy outcome. We recommend careful ultrasound monitoring in the presence of this ultrasonographic sign to exclude any other cause of increased intracranial pressure. To better understand the nature of these ultrasonographic signs, additional reports are deemed necessary. In fact in our case, as confirmed by histopathological examination, the fetal condition was extremely compromised due to failure of the fetal liver. Ductal plate malformation altered the liver structures causing hypoproteinemia and probably portal hypertension. These two conditions therefore explain the severe hydrops that compromised the fetal situation.

## Introduction

Our study analyzes a case of reverse end-diastolic flow in the middle cerebral artery (MCA) in a fetus at 26 weeks' gestation with a previous diagnosis of idiopathic ascites. This particular ultrasound pattern was associated with a rare liver abnormality known as ductal plate malformation (DPM). This condition is a rare malformation of intrahepatic bile ducts (IHBDs) due to an arrest or failure of epitheliomesenchymal inductive interactions. Immature bile ducts are subject to a progressive destructive cholangiopathy, resulting in a pattern of a more or less advanced fetal type of liver fibrosis [1]. The disequilibrium of the portobiliary system is often associated with autosomal recessive polycystic kidney disease or other fibrocystic malformation of the liver or kidneys, even if it has also been reported as an isolated entity [2] that is usually not identified by prenatal ultrasound examination. Only in rare cases of prenatal onset fetal liver cysts can be seen, and an ultrasound scan is the most useful method in showing the kidneys' anomalies associated with renal disease [3]. Reverse flow in the MCA is usually a transient event. The occurrence of this particular waveform can be explained by a few possible mechanisms that could alter cerebral blood circulation. Reverse flow could be a consequence of any elevated pressure condition outside or inside the brain. An elevation of external pressure can be due to mechanical compression of the fetal head by the transducer, which may induce a reverse flow that causes a high impedance to blood flow in the cerebral circulation [4]. External pressure may be increased also in the presence of an extended oligohydramnios or anhydramnios [5]. An increase in internal pressure can be due to the occurrence of hydrocephalus, cerebral edema, or cerebral hemorrhage. All of these pathological events can explain reverse flow in the MCA [6,7]. Reverse flow could also represent the extreme form of a brain-sparing mechanism before fetal death in case of intra-uterine growth restriction (IUGR) [8]. The other elements that can explain the reversal of flow in the MCA include abnormal fetal heart rate, the presence of tricuspid regurgitation, maternal drug effect, and temporary changes in fetal blood pressure after invasive intra-uterine procedures [9].

## Case presentation

**Figure 1:**
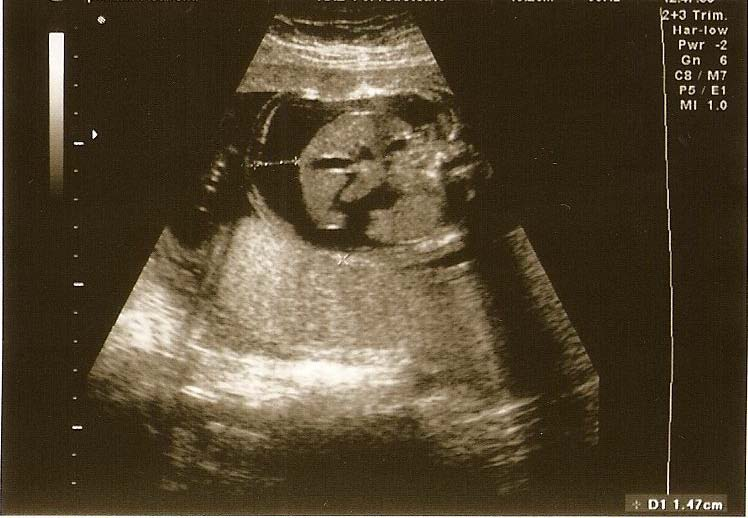
Sonogram showing fetal ascites

**Figure 2:**
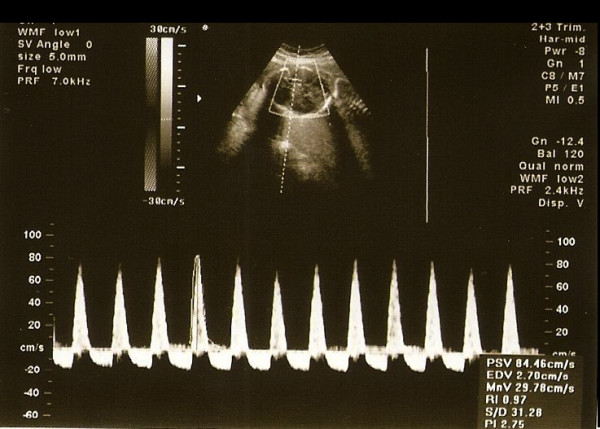
Sonogram showing reverse flow in the MCA

A 28-year-old Caucasian woman, gravida 4, para 1, was referred to our high-risk obstetric unit at 24 weeks' gestation for fetal ascites detected during a routine ultrasound examination. Her personal and family history did not reveal any pathology of note. Her first pregnancy ended with an intra-uterine death at 23 weeks' gestation caused by chorioamnionitis. In the second pregnancy, a live baby girl was delivered at 40 weeks' gestation. Four years later our patient had a spontaneous miscarriage. Our patient denied any fever, rash, cold symptoms, or joint pain before and during pregnancy. She did not refer to any vaginal bleeding. Laboratory tests for *Toxoplasma *and rubeola showed negative immunoglobulin M (IgM). An ultrasound examination performed during the consultation confirmed the presence of abdominal ascites (Figure 1). A borderline monolateral dilation of the cerebral ventricle was also seen. Biometry of the fetal limbs was below the mean in relation to gestational age, while cephalic measurements were normal. No other fetal anomalies were observed. Amniotic fluid was present in adequate quantity, but fetal movements were poor. For these reasons, the woman was admitted to our institution for close pregnancy monitoring. During hospitalization, her blood pressure and heart frequency were measured several times during the day, while cardiotocography was performed twice a day. Maternal blood pressure was normal, and there was no proteinuria. Investigation about the etiology of the fetal ascites were carried out, and a fetal paracentesis was also performed. Both parents had normal mean corpuscular erythrocyte volume with no sign of microcythemia and they both had positive blood group Rh without any pathologic antibodies. A routine blood laboratory assessment did not show any kind of abnormalities. Viral serology markers (cytomegalovirus IgG and IgM, parvovirus IgG and IgM) were negative in the maternal blood, and no viral genome was isolated in her amniotic fluid or the fetal ascites sample. The fetal karyotype was normal (46, XY). A Kleihauer-Betke test showed no evidence of fetal erythrocytes in the maternal circulation. Immunologic markers, lupus anticoagulant anticardiolipin, antinuclear, and anti-RO antibodies were negative; G6PD was also excluded. There was no evidence regarding the cause of fetal ascites (then considered non-immune or hydiopatic). Only a mild increase in inflammation indices was noted (VES 40, PCR 7.325); an antibiotic preventive treatment was performed for five days (sulbactam/ampicillin 1.5 mg three times daily, gentamicin 80 mg three times daily, metronidazole 500 mg three times daily). During hospitalization, detailed ultrasound scans were performed at least every two days to monitor the ascites and general condition of the fetus. Fetal middle cerebral artery peak systolic velocity (PSV) was measured to diagnose fetal anemia. Pulsatility index (PI) of either the umbilical artery (UA) or the MCA as well as the resistance index of uterine arteries were assessed to better evaluate the materno-fetal perfusion. Monitoring scans showed a deterioration of fetal condition. Paracentesis was performed at 25 weeks' gestation, but two days later the fetal ascites occurred again. A restriction of fetal growth and progressive reduction of amniotic fluid were also registered. The value of PSV in the MCA was borderline for moderate to severe anemia according to Mari's chart [10], whereas fetal Doppler ultrasound parameters were normal. At 27 weeks' gestation, a reverse end-diastolic flow in the MCA occurred (Figure 2). This abnormal waveform pattern persisted for all the ultrasound examinations, and it was not associated with other Doppler abnormalities (PI UA 1.15, PI MCA 1.54). The condition was interpreted as 'at risk', and our patient was submitted to closer monitoring. Fetal echocardiography showed cardiomegaly without significant abnormality of heart structures and a mild tricuspid regurgitation. Cardiotocography monitoring showed fetal bradycardia (fetal heart rate 100 beats per minute). Our patient was submitted to fetal magnetic resonance imaging to investigate in particular the brain edema, and this confirmed the presence of fetal ascites without showing any abnormality of fetal cerebral tissue or signs of hypoxia. Corticosteroid treatment (betamethasone 12 mg for two days) was started because of a high risk for preterm delivery. After one week, ultrasound parameters showed a severe decrease in fetal weight (<fifth percentile), an increase in fetal ascites, and subcutaneous edema. The reversed end-diastolic flow in the MCA persisted, and an increase of PI UA was detected. Alterations of ductus venosus waveform appeared, and an inversion of "a" wave was identified. The amniotic fluid index was below the mean for gestational age. The biophysical profile examination was unsatisfactory. The severe prognosis was explained to the couple, and an active intervention was ruled out. The fetus was in breech presentation at this time. Our patient was submitted to an emergency cesarean section at 28 weeks' gestation. A live baby boy in poor general condition was delivered. The Apgar score was one and five. Intubation was necessary, but despite neonatal intensive care, the baby died a few hours after birth. An autopsy revealed a rare malformation of IHBDs known as DPM. No other anomaly was identified.

## Discussion

Currently, Doppler ultrasound is widely used to study fetal circulation in both normal and pathological pregnancy. With regard to fetal cerebral circulation, quantitative and qualitative estimations of the MCA blood flow are usually performed. Blood flow impedance is studied in terms of PI in relation to gestational age; the qualitative assessment consists of a valuation of present, absent, or reverse blood flow during the diastole. Reverse end-diastolic flow in the MCA is usually a rare and transient event. In the majority of cases, the cause of this abnormal waveform pattern remains unknown even when increased cerebral pressure is considered. Sepulveda *et al*. [11] considered reverse flow in the MCA an agonal sign in the human fetus. They documented the first case in the literature of reverse end-diastolic flow in a severely growth-restricted fetus that appeared the day before fetal death. Leung *et al*. [12] documented this pattern waveform as a transient event in a severe IUGR fetus at 24 weeks' gestation. During the observation, the fetus was in breech presentation. Reverse flow in the MCA disappeared the day after, when the fetus modified its presentation in a cephalic one and did not come out again in the subsequent Doppler studies. These authors underlined that the fetus was in breech presentation when the reverse flow occurred in the MCA, whereas it was in cephalic presentation before and after. De Spirlet *et al*. [13] described a case of persistent reverse end-diastolic flow in a fetus with subdural hematoma due to severe thrombocytopenia. Respondek *et al*. [9] analyzed the outcome of 22 fetuses with reversal of diastolic flow in the MCA. The authors concluded that, in the majority of cases, this phenomenon is not related to fetal pathology, morbidity, and mortality. In their case, an intra-uterine death was observed in a fetus with a prolonged reverse flow in the MCA. In our case, we carefully minimized the transducer's pressure when the reversal of diastolic flow in the MCA appeared. We also used two different types of ultrasound equipment and either a trans-vaginal or trans-abdominal approach. No uterine contractions were observed during the examination. Despite all of these efforts, the waveform patterns persisted for the duration of ultrasound examination. The fetus also presented with bradycardia (<100 beats per minute) and a mild tricuspid regurgitation assessed during the echocardiography. This regurgitation was defined as not significant for the hemodynamic status of the fetal heart, and we assume that this was not correlated to the reversal of diastolic flow in the MCA. The peculiarity in this case was that in our patient, reverse flow in the MCA persisted for over one week and was not associated with reverse flow in the UA. However, it could be related to a deterioration of fetal condition.

## Conclusions

We agree with Sepulveda *et al*. [11] in considering reverse end-diastolic flow in the MCA a sign of poor fetal outcome. In fact, as confirmed by histopathological examination, the fetal condition in our case was extremely compromised by failure of the fetal liver. The DPM altered the liver structures, causing hypoproteinemia and probably portal hypertension. These two conditions can explain the severe hydrops that compromised the fetal condition. According to the literature, no ultrasound sign of liver disease can be seen on prenatal ultrasound examination [3]. To the best of our knowledge, there is only one case in the literature of fetal ascites associated with DPM [14]. In that case, the massive ascites appeared later in gestation (33 weeks), and the baby died at 36 days of life despite intensive neonatal care. Regarding our experience, isolated and persistent reverse flow in the MCA should be considered a marker of adverse pregnancy outcome. In this condition, we recommend close ultrasound monitoring to exclude any cause of increased intracranial pressure and to identify other signs of impending fetal adverse outcome. We recommend repeated ultrasound and Doppler assessments at not more than daily intervals. To better understand the nature of this ultrasound sign and its relation with poor fetal outcome, additional reports are deemed necessary.

## Abbreviations

DPM: ductal plate malformation; IHBD: intrahepatic bile duct; Ig: immunoglobulin; IUGR: intra-uterine growth restriction; MCA: middle cerebral artery; PI: pulsatility index; PSV: peak systolic velocity; UA: uterine artery

## Consent

Written informed consent was obtained from the patient for publication of this case report and accompanying images. A copy of the written consent is available for review by the Editor-in-Chief of this journal.

## Competing interests

The authors declare that they have no competing interests.

## Authors' contributions

AG contributed to the concept and design of the case report; AS contributed to the design of the case report, revisiting the manuscript critically for important intellectual content; VD contributed to the collection and interpretation of data and made a major contributor to writing the manuscript; GP contributed to the collection and interpretation of data and contributed to writing the manuscript; BM contributed to the interpretation of data; FB contributed to the interpretation of data; RLT contributed to the critical writing and revisited the intellectual content; MMA contributed to the critical writing and revisiting the intellectual content; FDG revisited the manuscript critically for important intellectual content. All authors read and approved the final manuscript.
